# Numerical hemolysis performance evaluation of a rotary blood pump under different speed modulation profiles

**DOI:** 10.3389/fphys.2023.1116266

**Published:** 2023-02-02

**Authors:** Feng Huang, Huan Lei, Shunv Ying, Yang Fu, Qipeng Li, Xiaodong Ruan

**Affiliations:** ^1^ School of Mechanical and Energy Engineering, Zhejiang University of Science and Technology, Hangzhou, China; ^2^ State Key Laboratory of Fluid Power and Mechatronic Systems, Zhejiang University, Hangzhou, China; ^3^ Stomatology Hospital, School of Stomatology, Zhejiang University School of Medicine, Clinical Research Center for Oral Diseases of Zhejiang Province, Key Laboratory of Oral Biomedical Research of Zhejiang Province, Cancer Center of Zhejiang University, Hangzhou, China

**Keywords:** rotary blood pump, hemolysis, numerical evaluation, speed modulation, computational fluid dynamics

## Abstract

**Introduction:** Speed modulation methods have been studied and even used clinically to create extra pulsation in the blood circulatory system with the assistance of a continuous flow rotary blood pump. However, fast speed variations may also increase the hemolysis potential inside the pump.

**Methods:** This study investigates the hemolysis performance of a ventricular assist rotary blood pump under sinusoidal, square, and triangular wave speed modulation profiles using the computational fluid dynamics (CFD) method. The CFD boundary pressure conditions of the blood pump were obtained by combining simulations with the pump’s mathematical model and a complete cardiovascular lumped parameter model. The hemolysis performance of the blood pump was quantified by the hemolysis index (HI) calculated from a Eulerian scalar transport equation.

**Results:** The HI results were obtained and compared with a constant speed condition when the blood pump was run under three speed profiles. The speed modulations were revealed to slightly affect the pump hemolysis, and the hemolysis differences between the different speed modulation profiles were insignificant.

**Discussion:** This study suggests that speed modulations could be a feasible way to improve the flow pulsatility of rotary blood pumps while not increasing the hemolysis performance.

## 1 Introduction

Rotary blood pumps (RBPs) have been an effective clinical approach to support blood circulation in patients with end-stage heart failure (Timms, 2011). Through the past decades, significant progress has been made on the RBPs, and they have already become the newest and most popular ventricular assist device (VAD). They are superior in smaller sizes and have better reliability than the first generation of volume displacement blood pumps. RBPs can be used as either a transitional treatment when waiting for a heart donor or permanent treatment that completely replaces the original heart.

Although RBPs have many advantages, their non-pulsatile flow pattern is non-physiological and may cause issues such as vital organ injury and vascular sclerosis (Hornick and Taylor, 1997; Alkan et al., 2007; Purohit et al., 2018). On the other hand, the long-time invariable continuous flow through the pump may also induce thrombus in flow stagnant areas inside the pump. To solve this problem, speed modulation methods have been proposed to increase the pulsatility of continuous flow RBPs (Shiose et al., 2010; Pirbodaghi et al., 2012; Amacher et al., 2014; Huang et al., 2014; Kumar et al., 2019). By periodically adjusting the pump speed, the flow pulsation through the blood pump could be increased that is helpful for pump washing and in reducing the risk of thrombosis, and at the same time enhancing the pulsation of the blood vascular system (Wang et al., 2021). Soucy et al. (2015) investigated three types of the most common pump speed modulation profiles, namely, co-pulsation, counter-pulsation, and low-frequency asynchronization, in a chronic ischemic heart failure bovine model, demonstrating that pump speed modulation increases pulsatility and improves cardiac function and end-organ perfusion. The HVAD (HeartWare, Miami Lakes, FL, United States) and HeartMate 3 (Abbott, St. Paul, MN, United States) blood pumps, as two of the most successful clinical blood pump products worldwide, have been tested for their speed modulation methods in clinical studies (Bourque et al., 2016; Kumar et al., 2019).

Along with the usage of speed modulation methods of RBPs comes more considerations such as their negative effects. Speed modulations force sharp variations in pump speed that significantly disturb the flow field inside the pump. As a result, shear stress is increased that damages the blood more. Wiegmann et al. (2019) recently investigated the effect of HeartMate 3 on flow fields using computational fluid dynamics (CFD) when operating in the “artificial pulse” speed modulation and found increased turbulence and total stress. Furthermore, Chen et al. (2019) carried out CFD simulations of HVAD under asynchronous speed modulation and reported no obvious increase in hemolysis results. However, until now, these relevant studies have been few, and only one single speed modulation type has been investigated in each of the aforementioned studies. The speed profile of a modulation method has a variety of types and on whether the basic sinusoidal, square, or triangular waveforms could be used as a speed profile (Pirbodaghi et al., 2012) has not been well revealed. There has been no report regarding the effects on the inner flow field and hemolysis under these differential speed modulation profiles.

One difficult issue in conducting CFD simulations for speed-modulated blood pumps has been in obtaining the boundary conditions. Chen et al. (2019) used experimentally recorded pressure waveforms from animal trials. However, speed adjustments of the blood pumps are restricted and some desired extreme speed variations cannot be acquired due to security concerns. Moreover, animal tests are too expensive to be carried out frequently. System simulation with cardiovascular and pump models could be an alternative way to provide the pressure or flow boundary conditions. In this case, a complete functional cardiovascular system model is desired.

In this study, a comprehensive comparative study on the hemolysis performance of the rotary blood pump under three different speed modulation profiles was conducted by combining CFD and cardiovascular system simulation methods. The time–course distribution of the hemolysis index (HI) inside the pump under speed modulation and constant speed conditions was obtained and compared. The pump’s outlet and HI average values revealed that hemolysis performance degeneration under speed modulation was very small, and there was no obvious hemolysis difference among the different speed modulation profiles. The main contributions of this work are highlighted as follows. First, the hemolysis performance under multiple speed modulation types of a specific rotary blood pump was evaluated with CFD for the first time. Second, based on the complete cardiovascular system model that included not only the circulatory system but also the baroreflex regulation, the pressure and flow waveforms are physiological and comparable to experimental results, which was qualified to provide the boundary condition for CFD simulations.

## 2 Materials and methods

### 2.1 Meshing of rotary blood pump

**FIGURE 1:**
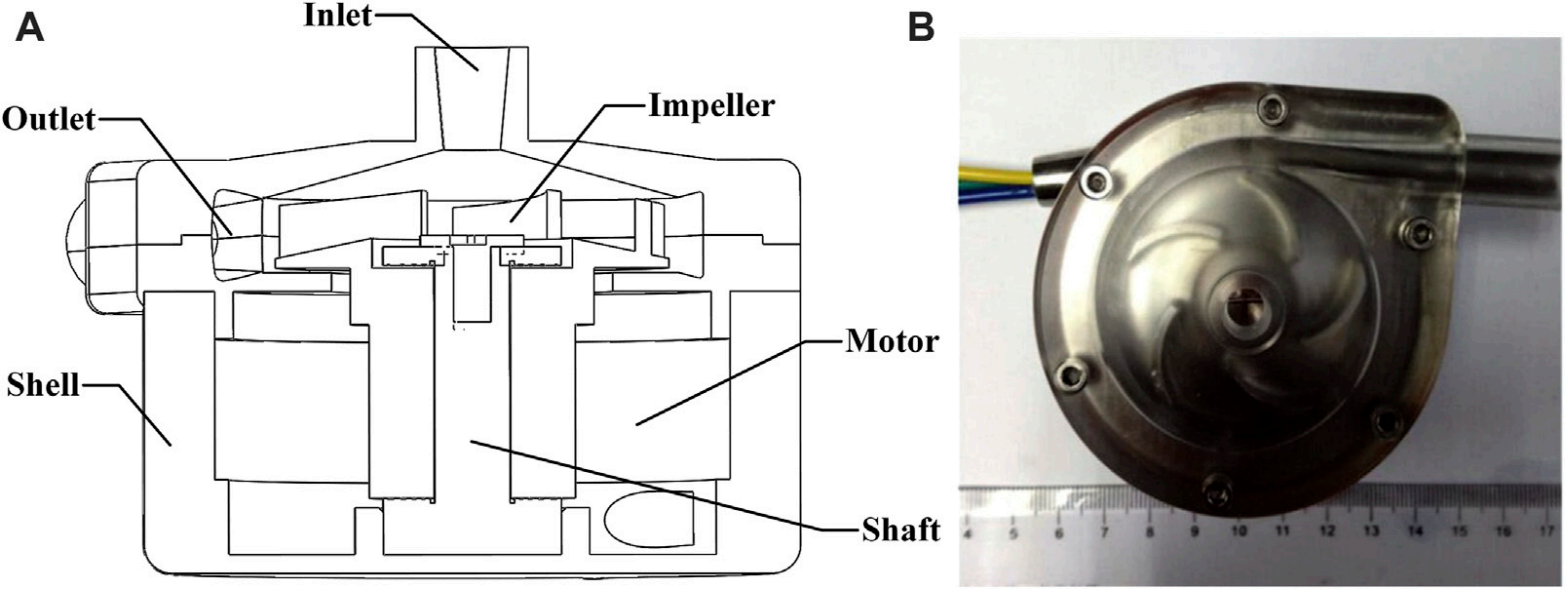
Structure and photo of the rotary blood pump. **(A)** Structure of the blood pump; **(B)** top-view photo of the blood pump.

The research object in this study is a self-developed centrifugal rotary blood pump by our group (Li et al., 2019). Figure 1A shows its structure, and Figure 1B shows the top-view photo of the pump. The pump has an 8-mm inner diameter outlet and a 6-mm inner diameter inlet. The impeller is designed as a semi-open type and has five blades. The pump’s rated working rotary speed is 3,000 rpm, at which the pump could deliver a blood flow of 5 L/min against a 100 mmHg pressure head.

**FIGURE 2:**
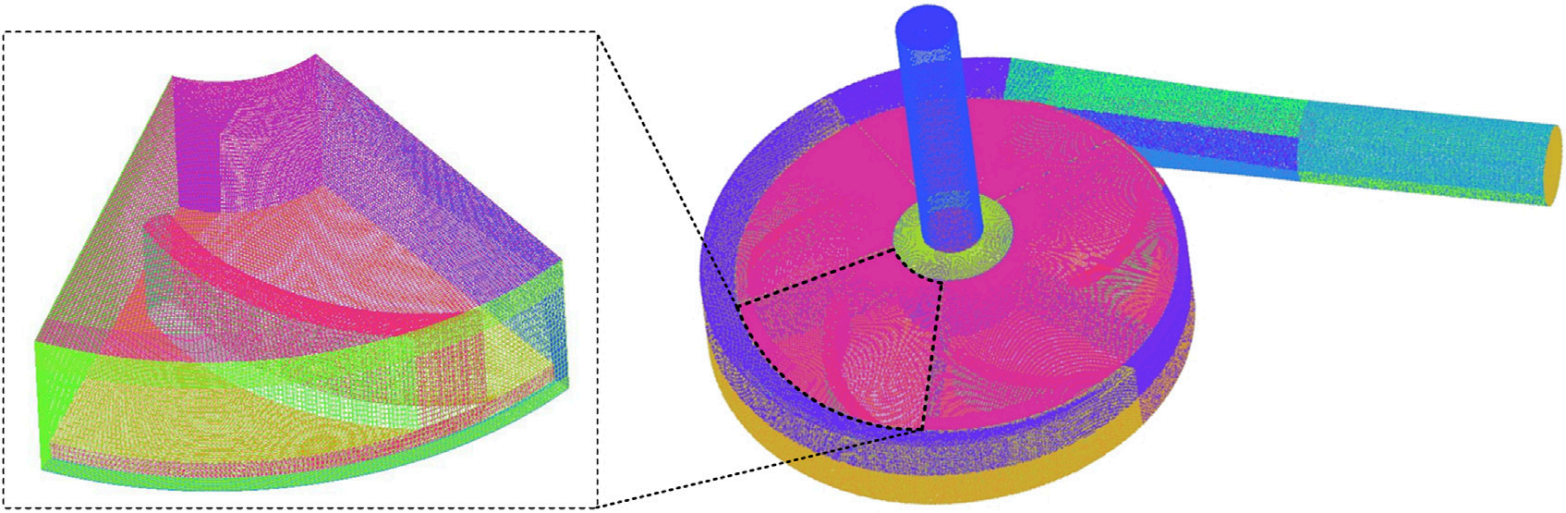
Meshing of the fluid domain of the blood pump.

The fluid domain of the blood pump is imported into the preprocessing software ICEM (ANSYS, Inc. Canonsburg, PA, United States) for meshing operations. To improve the simulation result, the fluid domain was divided into five parts, namely, inlet, outlet, blade region, impeller center region, and volute, with each part meshed separately. Local mesh refinements were applied to the pivotal blade region and narrow gaps between the pump housing and impeller blades. Structured hexahedral meshes were used for all the regions. A mesh-independent analysis was also conducted, and an element number increase of 30% only led to a 0.2% difference in the steady hydraulic result, demonstrating the validity of the mesh. At last, the total amount of mesh elements was selected as 5.66 million. Figure 2 shows the fluid domain meshing of the blood pump.

### 2.2 Computational fluid dynamics boundary conditions

The boundary conditions required in the CFD simulations were chosen as the inlet and outlet dynamic pressures of the blood pump. The pressures should be obtained with the interaction of the blood pump and blood circulatory system. It is difficult and unsafe to measure the real pressure data from a patient with a rotary blood pump under a non-clinically verified speed modulation operation. As a result, the system simulation data from a complete cardiovascular lumped parameter model incorporated with the mathematical model of our blood pump was obtained and used as the pressure boundary condition.

#### 2.2.1 Speed modulation profiles

**FIGURE 3:**
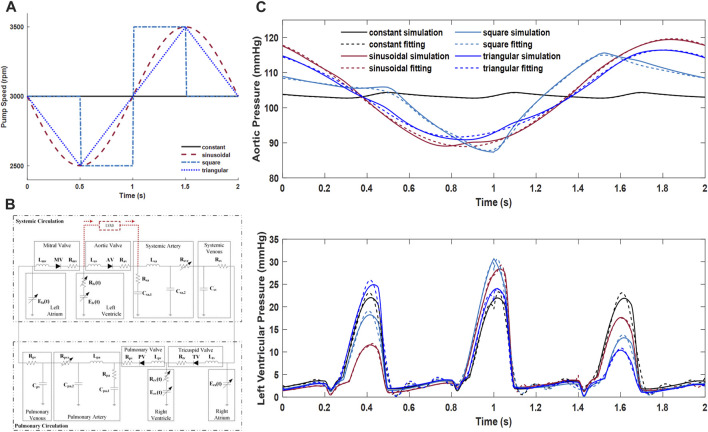
**(A)** Configuration of the speed modulation profiles. **(B)** Cardiovascular system model and its connection of the RBP. **(C)** Left ventricular and aortic pressures from system simulations and their piecewise function fitting results that were used as the CFD boundary conditions.

The three different types of speed modulation profiles, namely, sinusoidal, square, and triangular waves, are pending investigation. The constant speed condition was also included as the baseline for comparison. All the speed setups are shown in Figure 3A. The RBP was run at the constant speed condition under a fixed rotary speed of 3,000 rpm, while the other three speed setups enforced the RBP rotating around 3,000 rpm with an amplitude of 500 rpm. The period of the sinusoidal, square, and triangular waveforms was set to 2 s, which means that the speed modulations were asynchronized with the beating of the heart that is normally within 1 s. This asynchronization configuration is more realistic in terms of the actual speed response of general blood pumps.

#### 2.2.2 Cardiovascular system model

The lumped parameter mathematical model of the complete cardiovascular system used in this study was adopted from our previous study (Huang et al., 2019). Using the classical idea of an electric–fluid analog, Figure 3B depicts the complete cardiovascular system model and its connection with the blood pump. The RBP connected from the left ventricle to the systemic artery was severed as a left ventricular assist device (LVAD).

The cardiovascular system model is comprised of the systemic and pulmonary circulatory pathways. Concretely, the beating heart was modeled as a non-linear time-varying elastance model, with the elastance value representing its contractility. Both the ventricles and atria have their specific elastance values. The heart valves, preventing the backward flow of blood, were modeled as ideal diodes with infinite inverse resistances. The systemic or pulmonary arterial system applied the classic five-element Windkessel model, which describes the arterial system with resistance, compliance, and inertance and provides an efficient load to the heart over an entire frequency range of interest (Toy et al., 1985). The venous system, as the main reservoir of blood, was simply characterized by a lumped compliance along with resistance.

#### 2.2.3 Mathematical model of rotary blood pump

The RBP used in this study was modeled to have the co-simulation run. As only the hydraulics was concerned, the driven system of the pump was neglected during modeling. The dynamic hydraulic characteristic of the RBP is governed by an ordinary differential equation. Specifically, the pressure head ( H ) of the RBP depends on the second-order polynomial of the flow rate ( Q ) and its derivative ( $\dot{Q}$ ), as well as the square of rotary speed ( ω ).

$$H=a_{0}Q+a_{1}Q^{2}+a_{2}\dot{Q}+a_{3}\omega^{2}$$ (1)

where $a_{0}=-0.8875$ , $a_{1}=-0.00002726$ , $a_{2}=-0.07781$ , and $a_{3}=0.00001995$ are the coefficients that have been identified by the dynamic experimental pressure head and flow rate data under variable rotary speed using regression analysis (Li et al., 2019). In the abovementioned equation, the units for the flow rate, pressure head, and rotary speed are mL/s, mmHg, and rpm, respectively.

#### 2.2.4 System simulation results and boundary condition setting

System simulations when the pump runs according to the speed profiles as shown in Figure 3A were conducted using the Simulink/MATLAB software (MathWorks Inc., Natick, MA, United States). The left ventricular contractility was set to have only 20% of its full beating capacity to simulate a heart failure condition.

As the pump was with cannulation from the left ventricle to the aorta, the left ventricular and aortic pressures were obtained to serve as the boundary inlet and outlet conditions. To satisfy the CFX software configuration, the pressure data from system simulation were transformed to functions by curve fitting. Piecewise functions were applied to obtain a better fitting result. The R-square values of all the curve fitting results were better than 0.98, which was thought to be good fittings. The original waveforms of the left ventricular and aortic pressures under all speed conditions, and their fitting results with the piecewise functions that were used as the boundary conditions in the CFX software, are all depicted in Figure 3C

### 2.3 Hemolysis calculation

The hemolysis of the RBP was evaluated using the previously defined hemolysis index (HI) that was related to shear stress. In other words, although hemolysis could be caused by a variety of physical and chemical factors, only shear stress–induced blood damage was considered in this study. The shear stress vector obtained from the CFD-simulated flow field was first converted to scalar shear stress (SSS) by the following expression:

$$\sigma ={\left[\frac{1}{6}\underset{i\neq j}{\sum }{\left(\tau_{ii}-\tau_{jj}\right)}^{2}+\underset{i\neq j}{\sum }{\tau_{ij}}^{2}\right]}^{\frac{1}{2}}$$ (2)

where $\tau_{ij}$ is the Cartesian component of shear stress.

The HI was then calculated by solving a Eulerian scalar transport equation that had been validated in previous studies (Taskin et al., 2012; Li et al., 2019):

$$\left(\frac{\partial }{\partial t}+v\nabla \right)\left({HI}^{1/\alpha }\right)=S$$ (3)

where v is the velocity vector, and S is the source term, which is defined as

$$S=C^{1/\alpha }\sigma^{\beta /\alpha }$$ (4)

where $C=1.21\times 10^{-5}$ , α=0.747 , and β=2.004 are the empirical constants (Taskin et al., 2010).

By solving the aforementioned scalar transport equation during CFD simulation, the distribution of the HI inside the pump could be obtained. The inlet boundary condition for the HI was set to zero. Also, the mass-weighted average of the HI values at the pump outlet was regarded as the final hemolysis performance.

### 2.4 Numerical configuration

The CFD simulations were conducted with the commercial software CFX (ANSYS, Inc., Canonsburg, PA, United States). The shear stress transport (SST)–based k-ω model was adopted as the turbulence model. Blood, as the working fluid, was assumed to be an incompressible Newtonian fluid, with its density and viscosity set at 1,050 kg/m^3^ and 0.0035 Pa s, respectively.

The Multiple Reference Frame method was used for the rotating simulation. The dynamic and static interfaces between the rotating and static domains were set to a frozen rotor configuration in the CFX, and no slip model was applied to solid walls. The continuity and momentum governing equations were set down in both the rotating and stationary reference frames, which can be written as

$$\nabla ∙\left(\rho U\right)=0$$ (5)

$$\frac{\partial \left(\rho U\right)}{\partial t}+\nabla ∙\left(\rho U∙U\right)=-\nabla p+\nabla ∙\tau +S$$ (6)

where ρ is the density, U is the relative frame velocity, and τ is the stress tensor. S is the source term, which is 0 in the stationary reference frame and $S=-2\rho \omega \times U-\rho \omega \times \left(\omega \times r\right)$ in the rotating reference frame, where ω is the angular speed and r is the location vector.

To eliminate the initialization effect and guarantee the stable result of a flow field, simulation was first initialized over three whole cardiac cycles (2 s), and then three subsequent cardiac cycles were simulated as the stable result. The time step of all CFD simulations was set to 0.001 s. When the monitored pump flow was stabilized and the residual was below 10^−4^, the simulation was regarded as convergent.

Besides the transient simulations of the three speed modulation conditions, the constant speed simulation was also included for comparison. More importantly, the hydraulic result predicted by the CFD simulation under the constant speed condition was compared to the experimental pressure head and flow rate data that were used in the RBP modeling to validate the effectiveness of the CFD model.

## 3 Results

### 3.1 Verification of computational fluid dynamics model

**FIGURE 4:**
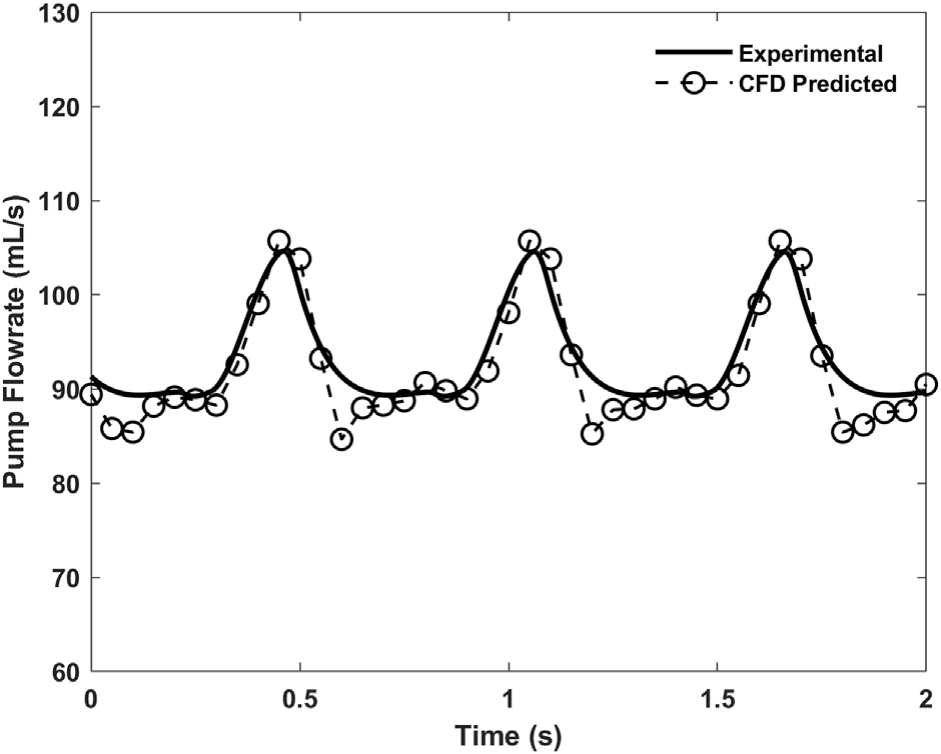
Time-varying pump flow rates predicted by CFD simulation and the corresponding experimental data under the 3,000 rpm constant speed condition.

The time-varying pump flow rates predicted by CFD simulation under the constant speed condition are plotted in Figure 4, where the corresponding experimental flow rate data those were used in the RBP modeling are also depicted. A good agreement with the root mean square error (RMSE) of about 2.54 mL/s was found between them, which demonstrates well the validity of the CFD model. It is noted that although the pump speed is constant, the pump flow is still pulsatile due to the influence of the heart beating, and it is a transient simulation verification.

### 3.2 Scalar shear stress distribution

**FIGURE 5:**
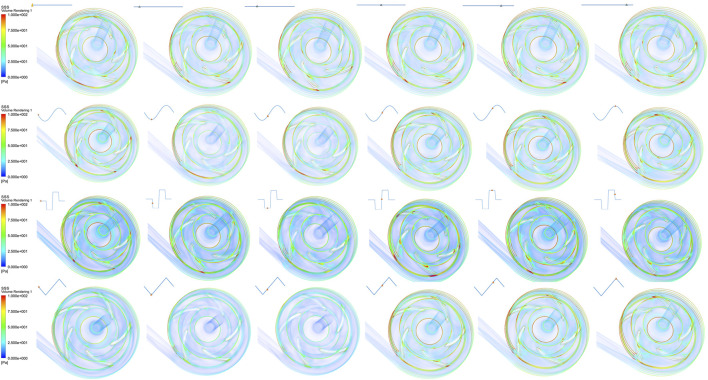
Scalar shear stress (SSS) distributions under constant speed and sinusoidal, square, and triangular speed modulation conditions.

Scalar shear stress (SSS) is a key factor used in hemolysis calculation. The distributions of SSS under constant, sinusoidal, square, and triangular speed modulation conditions are depicted in Figure 5. The highest SSS occurs on the surface of the impeller blades and near the pump housing, where a value of about 100 Pa is found. According to the previous report (Chen et al., 2016), SSS that is much greater than 10 Pa belongs to the non-physiological category and would cause platelet activation and hemolysis. Therefore, these regions of the pump are potentially dangerous areas for blood damage. However, the blood in most other regions inside the pump is exposed to a relatively low SSS condition and has a low risk of hemolysis.

### 3.3 Hemolysis performance

**FIGURE 6:**
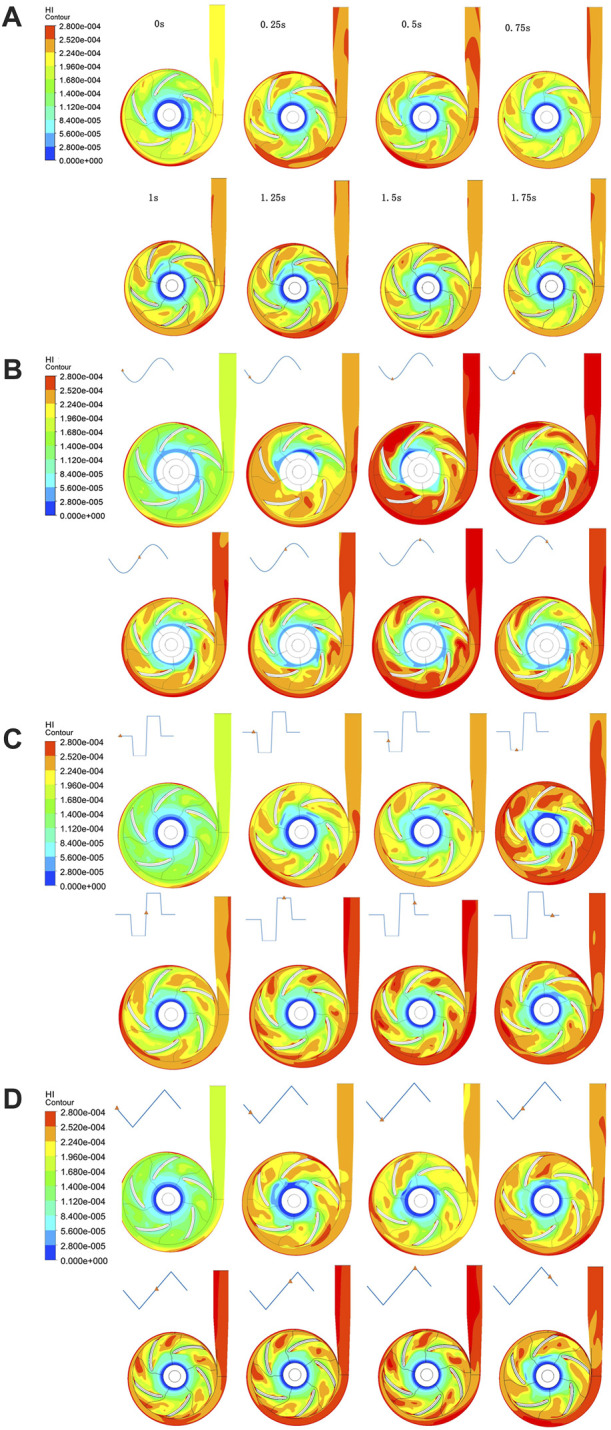
Time–course contour maps of the HI distribution on the radial cut plane of the pump under **(A)** constant, **(B)** sinusoidal, **(C)** square, and **(D)** triangular speed modulation conditions.

The hemolysis performance of the pump is denoted by the HI results calculated using the Eulerian scalar transport Eq. 3 based on flow field variables. The simulated HI distributions during one speed modulation cycle are shown in Figures 6B–D for sinusoidal, square, and triangular speed modulation conditions, respectively. For comparison, Figure 6A also shows the HI distribution when the pump speed is constant. As indicated by the figure, high HI occurs in the rotating regions of the impeller blades and at the pump’s outlet. With speed modulation, regardless of which speed profile, the HI at the end time of the simulated cycle was found to be greater than that of the constant speed condition. It can be seen from the comparison between constant speed and speed modulation results that the speed variation indeed affects the HI values at the pump’s outlet. Sudden acceleration increases pump hemolysis. It has been noted that even under the constant speed condition, due to the residual cardiac function, the HI distribution still changes mildly with time.

**FIGURE 7:**
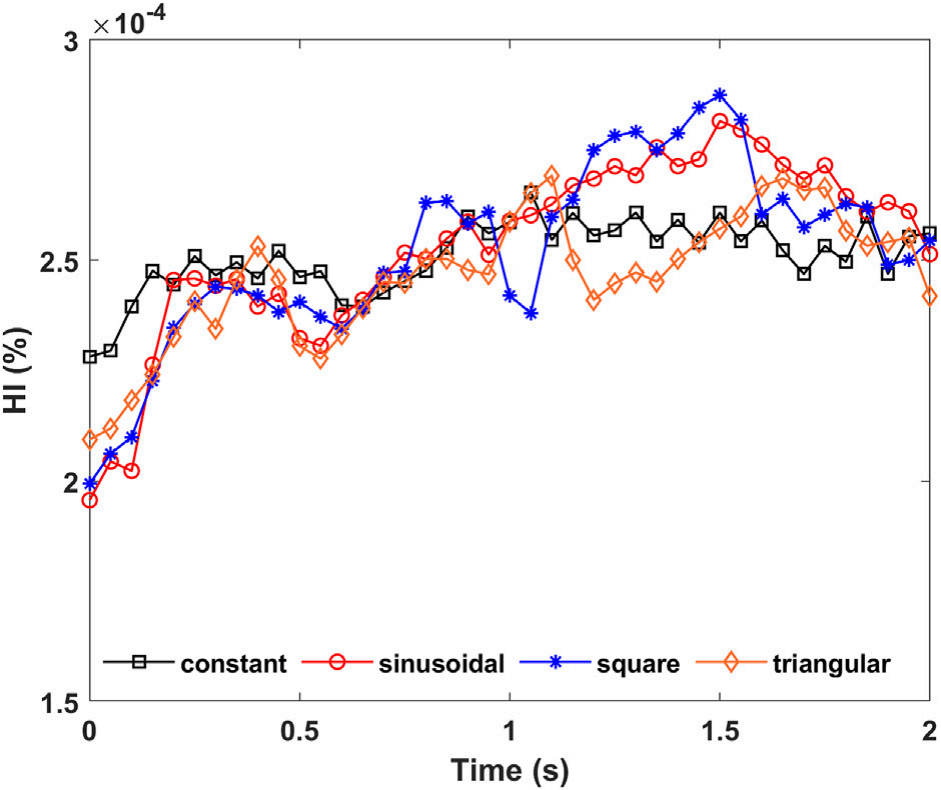
Time–course mass-weighted average HI values at pump’s outlet under constant, sinusoidal, square, and triangular speed modulation conditions.

Figure 7 shows the plotted curves of the mass-weighted average HI values at the pump’s outlet under constant speed and speed modulation conditions. There is a consistent trend between the average HI value and pump speed. During all the modulations, the pump speed reached its lowest near 0.5 s, which corresponds to a local minimum HI value, while the HI value reached a maximum as the pump speed increased to 3,500 rpm near 1.5 s. When compared to the constant speed condition, the HI values under speed modulations have a larger fluctuation range. Considering total hemolysis during the 2 s simulation time, the mass-weighted average HI values at the pump’s outlet were averaged with time and the calculated results for all the conditions were around 2.5 × 10^–6^, which are all within the acceptable range. The differences in the time-averaged HI values are within 2% and thought to be negligible, indicating no obvious risk for pump hemolysis with different pump speed modulation profiles. However, the triangular speed modulation seemed to have, although very slightly, a better hemolysis performance than the other speed modulation methods.

## 4 Discussion

Hemolysis is one key performance of a blood pump. Numerical hemolysis evaluations using a CFD method have been adopted by many researchers in the design optimization of a pump (Chen et al., 2019; Li et al., 2019; Wiegmann et al., 2019; Wu et al., 2021). It is a good complement to *in vivo* and *in vitro* hemolysis tests. In this research, we conducted a comprehensive comparative study on the numerical hemolysis performance of a RBP under constant speed and three different speed modulation profiles by using CFD simulations. The results could be a good reference for the actual clinical speed operation of the RBP.

Speed modulation has become an important topic in the clinical use of modern RBPs. Both the HVAD and HeartMate 3 devices have adopted their own speed modulation methods called Lavare Cycle and artificial pulse, respectively, and have conducted initial clinical trials. Besides, more speed modulation types may be developed in the future. It has become necessary to reveal the hemolysis performance under various modulation conditions. Till now, there have been only very few research studies (Chen et al., 2019; Wiegmann et al., 2019) reported, and the speed profile assessed is single. Considering a more complicated speed modulation profile could be composed of the basic profiles—basic sinusoidal, square, and triangular waves—which were chosen to be the evaluated speed profiles in this study. By referring to the speed modulation amplitude in the Lavare Cycle of the same centrifugal type of RBPs and combining the hydraulic characteristics of our pump, a larger amplitude of 500 rpm was set in the simulation. According to the experimental response of a blood pump (Shiose et al., 2010), these rotary speed variations are achievable in actual applications, but with subtle differences due to a specific motor’s response.

CFD has already become a powerful technology in the hydraulic design of blood pumps. It could also be used for numerical hemolysis evaluation. However, the accuracy of the CFD-based hemolysis prediction is still controversial. It fails to estimate the absolute values of *in vivo* hemolysis. The key hemolysis model is still empirical, such as the power-law model (Giersiepen et al., 1990) used in our study. Although many researchers are devoted to improving the hemolysis model (Li et al., 2014; Wu et al., 2019), the result is still unsatisfactory. However, our study is not aimed to predict absolute hemolysis but compare hemolysis differences among different speed modulations. In this sense, it is reasonable to adopt the popular power-law hemolysis model. On the other hand, the parameters of the power-law model have different values for different species (Ding et al., 2015). In this study, we adopted the values measured from animal blood by Taskin et al. (2010), which are popularly used in many research studies. The predicted absolute hemolysis index will be different from that obtained for the parameter values measured from the human blood. However, as just mentioned, for a comparative study, absolute hemolysis is not the primary concern, and the parameter values used in this study are adequate.

Shear stress is closely related to hemolysis of the blood pump. During speed modulations, an increase in the pump’s rotary speed causes a greater magnitude of the highest SSS value, and slightly more volume of the non-physiological SSS regions. When compared with the constant speed condition, there is no obvious difference in the average volume of high-value SSS found for any speed modulation condition as seen in Figure 5. Besides, the volume difference of non-physiological SSS regions is also statistically insignificant among the three modulation methods. These results indicate that the speed modulations used in this research only affect the non-physiological SSS regions of the blood pump very slightly.

Consistent with the shear stress result, there is no significant hemolysis difference among the different speed modulation profiles in this study. The result also indicates no significant difference of the average exposure times among the different modulations. Similar results have also been reported in previous reports (Chen et al., 2019; Wang et al., 2019). The possible reason for this might be related to the same change in amplitude of the pump speed. Although different speed modulation waveforms bring different flow fields, under the same waveform amplitude, the overall effect difference is very small. In addition, the amplitude used in this study might not be large enough to cause significant difference in the flow field and hemolysis. Nevertheless, simulations with larger amplitudes are not necessary because large pump speed changes are rare in clinical trials.

Boundary conditions are necessary for CFD simulations. In simple scenarios, such as the steady running mode of RBPs with constant speed and without connection to the blood circulatory system, it is feasible to obtain the boundary pressure or flow conditions by simple *in vitro* experiments. However, this would become difficult when the pump interacts with the natural heart and blood circulatory system, because in this case, *in vivo* experiments or at least *in vitro* experiments with a well-designed and full-featured mock circulatory system (Huang et al., 2013) must be required. Moreover, if the pump has various speed operations, the experiments would become more difficult. To avoid *in vivo* experiments, in our study, system simulations using a validated cardiovascular system model coupled with the blood pump model were conducted using the Simulink/MATLAB software to obtain the inlet and outlet pressures of the pump for setting the boundary conditions. The system simulations were proven to replicate similar physiological waveforms when a blood pump was implanted (Huang et al., 2018, 2019), which is adequate for comparative CFD studies. This provides a convenient and effective means for obtaining the boundary conditions. With easy parameter modifications, pressure and flow conditions under various operation modes of the blood pump can be obtained.

It has to be noted that there are some limitations in this study. First, the hemolysis evaluation is numerical and lacks experimental validation. Inspired by this study, it is recommended to have some clinical hemolysis tests of the blood pumps under speed modulations. Second, the boundary pressure conditions set in the CFD simulations are fitting functions based on cardiovascular system simulations, which have certain deviations from the clinical waveforms. Third, as mentioned above, it is a comparative study based on the empirical power-law hemolysis model and, in the future, demands the development of a more accurate hemolysis model to support the numerical hemolysis evaluation. Besides, hemolysis induced by the clearance of the magnetic in the pump is ignored in the CFD simulation. As the result suggests non-obvious hemolysis differences among the three speed modulation profiles, the selection of the speed modulation profile may be considered with other factors such as the required flow pulsation amplitude or the actual speed response of the blood pump. In further work, more speed modulation modes and types of RBPs may be included to obtain a more complete hemolysis assessment.

## 5 Conclusion

In this research, a comprehensive comparative study on the hemolysis performance of the rotary blood pump under constant speed and three different speed modulation profiles was carried out using CFD simulations. The hemolysis performance of the pump was qualitatively evaluated with shear stress distribution and quantitatively assessed by the mass-weighted average HI values at the outlet of the blood pump. It has been revealed that the hemolysis differences among the different speed modulation profiles are non-obvious. This study suggests that the speed modulation method can be a feasible operation to improve flow pulsatility of the rotary blood pump while not increasing hemolysis.

## Data Availability

The original contributions presented in the study are included in the article/supplementary material; further inquiries can be directed to the corresponding authors.

## References

[B1] Alkan T. Akçevin A. Ündar A. Türkoğlu H. Paker T. Aytaç A. (2007). Benefits of pulsatile perfusion on vital organ recovery during and after pediatric open heart surgery. ASAIO J. 53, 651–654. 10.1097/MAT.0b013e31814fb506 18043139

[B2] Amacher R. Ochsner G. Schmid Daners M. (2014). Synchronized pulsatile speed control of turbodynamic left ventricular assist devices: Review and prospects. Artif. Organs 38, 867–875. 10.1111/aor.12253 24404879

[B3] Bourque K. Cotter C. Dague C. Harjes D. Dur O. Duhamel J. (2016). Design rationale and preclinical evaluation of the HeartMate 3 left ventricular assist system for hemocompatibility. ASAIO J. 62, 375–383. 10.1097/MAT.0000000000000388 27195742

[B4] Chen Z. Jena S. K. Giridharan G. A. Sobieski M. A. Koenig S. C. Slaughter M. S. (2019). Shear stress and blood trauma under constant and pulse-modulated speed CF-vad operations: CFD analysis of the HVAD. Med. Biol. Eng. Comput. 57, 807–818. 10.1007/s11517-018-1922-0 30406881 PMC6450749

[B5] Chen Z. Mondal N. K. Ding J. Koenig S. C. Slaughter M. S. Wu Z. J. (2016). Paradoxical effect of nonphysiological shear stress on platelets and von Willebrand factor. Artif. Organs 40, 659–668. 10.1111/aor.12606 26582038 PMC4871771

[B6] Ding J. Niu S. Chen Z. Zhang T. Griffith B. P. Wu Z. J. (2015). Shear-induced hemolysis: Species differences. Artif. Organs 39, 795–802. 10.1111/aor.12459 25899978

[B7] Giersiepen M. Wurzinger L. J. Opitz R. Reul H. (1990). Estimation of shear stress-related blood damage in heart valve prostheses - *in vitro* comparison of 25 aortic valves. Int. J. Artif. Organs 13, 300–306. 10.1177/039139889001300507 2365485

[B8] Hornick P. Taylor K. (1997). Pulsatile and nonpulsatile perfusion: The continuing controversy. J. Cardiothorac. Vasc. Anesth. 11, 310–315. 10.1016/S1053-0770(97)90100-2 9161899

[B9] Huang F. Gou Z. Fu Y. (2019). Preliminary evaluation of a predictive controller for a rotary blood pump based on pulmonary oxygen gas exchange. Proc. IMechE Part H. J. Eng. Med. 233, 267–278. 10.1177/0954411918823035 30760162

[B10] Huang F. Gou Z. Fu Y. Ruan X. (2018). Effects on the pulmonary hemodynamics and gas exchange with a speed modulated right ventricular assist rotary blood pump: A numerical study. Biomed. Eng. Online 17, 142. 10.1186/s12938-018-0591-4 30342521 PMC6195961

[B11] Huang F. Ruan X. Fu X. (2014). Pulse-pressure-enhancing controller for better physiologic perfusion of rotary blood pumps based on speed modulation. ASAIO J. 60, 269–279. 10.1097/MAT.0000000000000059 24614360

[B12] Huang F. Ruan X. Zou J. Qian W. Fu X. (2013). A fast building and effective hydraulic pediatric mock circulatory system for the evaluation of a left ventricular assist device. ASAIO J. 59, 575–585. 10.1097/MAT.0b013e3182a78e08 24088901

[B13] Kumar J. Elhassan A. Dimitrova G. Essandoh M. (2019). The Lavare cycle: A novel pulsatile feature of the HVAD continuous-flow left ventricular assist device. J. Cardiothorac. Vasc. Anesth. 33, 1170–1171. 10.1053/j.jvca.2018.11.029 30630656

[B14] Li H. Gou Z. Huang F. Ruan X. Qian W. Fu X. (2019). Evaluation of the hemolysis and fluid dynamics of a ventricular assist device under the pulsatile flow condition. J. Hydrodyn. 31, 965–975. 10.1007/s42241-018-0154-y

[B15] Li H. Ruan X. Qian W. Fu X. (2014). Numerical estimation of hemolysis from the point of view of signal and system. Artif. Organs 38, 1065–1075. 10.1111/aor.12294 24721170

[B16] Pirbodaghi T. Axiak S. Weber A. Gempp T. Vandenberghe S. (2012). Pulsatile control of rotary blood pumps: Does the modulation waveform matter? J. Thorac. Cardiovasc. Surg. 144, 970–977. 10.1016/j.jtcvs.2012.02.015 22418246

[B17] Purohit S. N. Cornwell W. K. Pal J. D. Lindenfeld J. A. Ambardekar A. V. (2018). Living without a pulse: The vascular implications of continuous-flow left ventricular assist devices. Circ. Hear. Fail. 11, e004670. 10.1161/CIRCHEARTFAILURE.117.004670 PMC6007027 29903893

[B18] Shiose A. Nowak K. Horvath D. J. Massiello A. L. Golding L. A. R. Fukamachi K. (2010). Speed modulation of the continuous-flow total artificial heart to simulate a physiologic arterial pressure waveform. ASAIO J. 56, 403–409. 10.1097/MAT.0b013e3181e650f8 20616704 PMC2933186

[B19] Soucy K. G. Giridharan G. A. Choi Y. Sobieski M. A. Monreal G. Cheng A. (2015). Rotary pump speed modulation for generating pulsatile flow and phasic left ventricular volume unloading in a bovine model of chronic ischemic heart failure. J. Hear. Lung Transpl. 34, 122–131. 10.1016/j.healun.2014.09.017 25447573

[B20] Taskin M. E. Fraser K. H. Zhang T. Gellman B. Fleischli A. Dasse K. A. (2010). Computational characterization of flow and hemolytic performance of the UltraMag blood pump for circulatory support. Artif. Organs 34, 1099–1113. 10.1111/j.1525-1594.2010.01017.x 20626739

[B21] Taskin M. E. Fraser K. H. Zhang T. Wu C. Griffith B. P. Wu Z. J. (2012). Evaluation of Eulerian and Lagrangian models for hemolysis estimation. ASAIO J. 58, 363–372. 10.1097/MAT.0b013e318254833b 22635012

[B22] Timms D. (2011). A review of clinical ventricular assist devices. Med. Eng. Phys. 33, 1041–1047. 10.1016/j.medengphy.2011.04.010 21665512

[B23] Toy S. M. Melbin J. Noordergraaf A. (1985). Reduced models of arterial systems. IEEE Trans. Biomed. Eng. 32, 174–176. 10.1109/TBME.1985.325439 3997173

[B24] Wang Y. Peng J. Qin K. Rodefeld M. D. Luan Y. Giridharan G. A. (2021). Control strategy to enhance pulmonary vascular pulsatility for implantable cavopulmonary assist devices: A simulation study. Biomed. Signal Process. Control 70, 103008. 10.1016/j.bspc.2021.103008

[B25] Wang Y. Shen P. Zheng M. Fu P. Liu L. Wang J. (2019). Influence of impeller speed patterns on hemodynamic characteristics and hemolysis of the blood pump. Appl. Sci. 9, 4689. 10.3390/app9214689

[B26] Wiegmann L. Thamsen B. de Zélicourt D. Granegger M. Boës S. Schmid Daners M. (2019). Fluid dynamics in the HeartMate 3: Influence of the artificial pulse feature and residual cardiac pulsation. Artif. Organs 43, 363–376. 10.1111/aor.13346 30129977

[B27] Wu P. Gao Q. Hsu P.-L. (2019). On the representation of effective stress for computing hemolysis. Biomech. Model. Mechanobiol. 18, 665–679. 10.1007/s10237-018-01108-y 30604300

[B28] Wu P. Huo J. Dai W. Wu W. T. Yin C. Li S. (2021). On the optimization of a centrifugal maglev blood pump through design variations. Front. Physiol. 12, 699891. 10.3389/fphys.2021.699891 34220556 PMC8249853

